# Dexmedetomidine Versus Nalbuphine as Adjuvants to 0.5% Ropivacaine in Ultrasound-Guided Supraclavicular Brachial Plexus Block: A Prospective Randomised Controlled Trial

**DOI:** 10.7759/cureus.86913

**Published:** 2025-06-28

**Authors:** Sunny Aggarwal, Priyanka Dev, Raju Shakya, Bhupendra Singh

**Affiliations:** 1 Anaesthesiology, North Eastern Indira Gandhi Regional Institute of Health and Medical Sciences (NEIGRIHMS), Shillong, IND; 2 Anaesthesiology, All India Institute of Medical Sciences, Raebareli, Raebareli, IND

**Keywords:** analgesia, brachial plexus block, dexmedetomidine, nalbuphine, ultrasound guided regional anesthesia

## Abstract

Background: Dexmedetomidine and nalbuphine are used as adjuvants to local anesthetics in regional anesthesia. This study aims to compare their efficacy and safety in supraclavicular brachial plexus block.

Methods: In this prospective, double-blind randomized study, 60 patients undergoing upper limb surgery were divided into two groups. Group D received ropivacaine with dexmedetomidine, and Group N received ropivacaine with nalbuphine. Parameters such as onset and duration of sensory and motor block, sedation, rescue analgesia requirement, hemodynamic variables and side effects were recorded.

Results: Group D demonstrated significantly faster onset and prolonged duration of sensory and motor blocks. Time to first rescue analgesia was significantly longer and the number of rescue doses was fewer in Group D. Sedation scores were higher in the early postoperative period in Group D. Bradycardia and hypotension were more frequent in Group D, while Group N exhibited stable hemodynamics but experienced higher incidence of nausea and pruritus compared to Group D.

Conclusion: Dexmedetomidine provides superior block characteristics and prolonged analgesia but with more hemodynamic effects. Nalbuphine is safer hemodynamically but with more nausea and pruritus.

## Introduction

Brachial plexus block (BPB) is a widely utilized regional anesthesia technique for upper limb surgeries, offering benefits such as effective analgesia, reduced opioid consumption, and minimized side effects associated with general anesthesia [1]. To enhance the efficacy and prolong the duration of BPB, various adjuvants are co-administered with local anesthetics. Among these, dexmedetomidine and nalbuphine have garnered attention for their potential to improve block characteristics and postoperative analgesia.

Dexmedetomidine, an α₂-adrenergic receptor agonist, is known for its sedative, anxiolytic, and analgesic properties. When used as an adjuvant in BPB, dexmedetomidine has been shown to prolong the duration of both sensory and motor blocks, as well as extend postoperative analgesia. However, its use is sometimes associated with side effects such as bradycardia and hypotension [2].

Nalbuphine, a synthetic opioid with mixed κ-agonist and μ-antagonist properties, has been explored as an adjuvant in regional anesthesia. Studies [3,4] have indicated that nalbuphine can enhance the quality of analgesia and prolong the duration of sensory blockade without significant adverse effects. In a comparative study, nalbuphine was found to be a better adjuvant than dexmedetomidine in supraclavicular brachial plexus block among young patients, offering longer block duration and fewer side effects [5].

Despite these findings, there remains a need for further research to directly compare the efficacy and safety profiles of dexmedetomidine and nalbuphine as adjuvants in BPB. Understanding their comparative effects on block onset time, duration, postoperative analgesia, and associated side effects is crucial for optimizing anesthetic management in upper limb surgeries.

This study aims to evaluate and compare the effects of dexmedetomidine and nalbuphine when used as adjuvants to ropivacaine in ultrasound-guided supraclavicular brachial plexus block. The primary focus is on assessing block characteristics, duration of postoperative analgesia, and the incidence of any adverse effects associated with each adjuvant.

## Materials and methods

This was a prospective, double-blind, randomized controlled study conducted at All India Institute of Medical Sciences, Raebareli, in a six-month period from October 2024 to March 2025 after obtaining approval from the Institutional Ethics Committee (Approval number 2021-13-IMP-1). The study was registered with the Clinical Trials Registry (number CTRI/2025/03/082843).

A total of 60 patients, aged between 18 and 60 years, belonging to American Society of Anesthesiologists (ASA) physical status I or II, scheduled for elective upper limb surgery under supraclavicular brachial plexus block between October 2024 to March 2025 were included. Patients with known allergy to local anesthetics or study drugs, coagulation disorders, infection at the injection site, chronic analgesic use and significant cardiovascular, hepatic or renal impairment (ASA 3 and above) were excluded. 

Patients were randomized into two groups using a computer-generated random number table. Group allocation was concealed in sequentially numbered, opaque, sealed envelopes. Patients in Group D (Dexmedetomidine) received 26 mL of 0.5% ropivacaine + 1 mL (100 µg) dexmedetomidine + 8 mL normal saline while those in Group N (Nalbuphine) received 26 mL of 0.5% ropivacaine + 1 mL (10 mg) nalbuphine + 8 mL normal saline. The total volume for both groups was standardized to 35 ml. Drug preparation was performed by an anesthesiologist not involved in the procedure or outcome assessment. The patient, the anesthesiologist performing the block and the observer collecting intraoperative and postoperative data were blinded to group allocation.

Under aseptic precautions, all patients were administered a supraclavicular brachial plexus block using USG-guided landmarks. Standard monitoring (heart rate, blood pressure, SpO₂, ECG, and respiratory rate) was carried out during the procedure and postoperatively at regular intervals. Sedation was assessed using the Ramsay Sedation Scale. Pain scores were recorded using the Visual Analogue Scale (VAS).

Primary outcomes measured were onset and duration of sensory and motor block. Secondary outcomes measured were hemodynamic changes (heart rate, mean arterial pressure, respiratory rate), time to first rescue analgesia, number of rescue doses in 12 hours and incidence of side effects (hypotension with mean arterial pressure less than 65 mmHg over the duration of block, bradycardia with heart rate less than 60 per minute over the duration of block, itching and nausea in first 24 hours after drug administration).

Sensory block was evaluated using pinprick stimulation to assess pain and a wisp of cotton to assess touch sensation. The evaluation was conducted over the C5-T1 dermatomes on forearm and hand, using a three-point scale: Score 0: Presence of pain sensation; Score 1: Absence of pain but presence of touch sensation; Score 2: Absence of both pain and touch sensation.

Motor block was assessed using a Modified Bromage scale for the upper extremities: Score 0: No block - complete flexion of both arm and forearm; Score 1: Partial block - complete forearm flexion with partial arm flexion; Score 2: Almost complete block - inability to flex the arm and reduced ability to flex the forearm; Score 3: Complete block - inability to flex both the arm and forearm.

Both sensory and motor blocks were assessed at one-minute intervals for the first 30 minutes following drug administration. Onset of sensory block was defined as time from the completion of drug administration to the achievement of a score of 2 across all C5-T1 dermatomes. Duration of sensory block was defined as time from the completion of drug administration to the return of normal sensation (score 0) in all evaluated dermatomes. Onset of motor block was defined as time from completion of drug administration to complete loss of motor function (score 3 on the Modified Bromage scale). Duration of motor block was defined as time from drug administration to full recovery of motor function (score 0 on the Modified Bromage scale). Duration of analgesia was defined as time from the end of drug administration to the first request for rescue analgesia. Rescue analgesia in the form of injection tramadol 50 mg slow i.v. was administered.

Data was analyzed using SPSS Statistics version 30 (IBM Corp., Armonk, NY, USA). Quantitative variables were compared using independent t-tests and qualitative variables were analyzed using the Chi-square test. A p-value < 0.05 was considered statistically significant.

## Results

**Figure 1:**
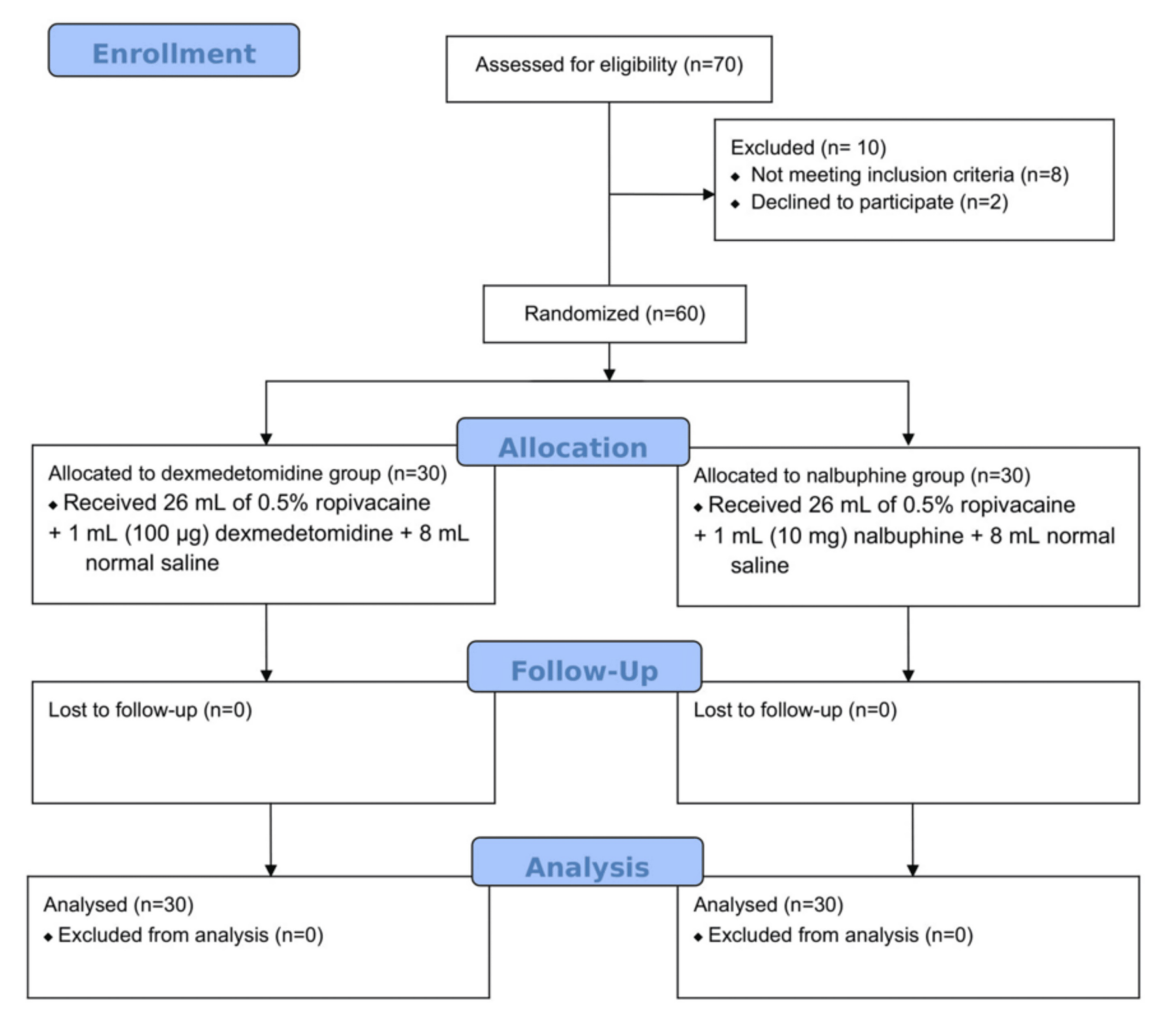
Consolidated Standards of Reporting trials (CONSORT) flow diagram

A total of 60 patients (30 in each group) received either dexmedetomidine or nalbuphine as adjuvant to 0.5% ropivacaine and finished the final analysis (Figure 1). There were no statistically significant differences between the demographic variables in two groups (Table 1).

**Table 1:** Baseline demographics of the two groups. ASA: American Society of Anesthesiologists

Parameter	Group D (Dexmedetomidine)	Group F (Fentanyl)	p-value
Age (years)	38.4 ± 10.2	39.6 ± 9.8	0.63
Sex (M/F)	18/12	17/13	0.79
Weight (kg)	65.3 ± 8.1	66.1 ± 7.9	0.58
Height (cm)	164.2 ± 6.5	165.5 ± 6.8	0.42
ASA I/II	22/8	21/9	0.77
Controlled Hypertensive patient(s)	6	8	0.57
Controlled Diabetic patient(s)	4	5	1.0

There was a significant difference between onset and duration of sensory and motor blocks and timing and requirement of number of rescue analgesia (injection tramadol 50 mg i.v.) between the two groups (using independent t-test) (Table 2).

**Table 2:** Block characteristics and rescue analgesia requirements in the two groups.

Parameter	Dexmedetomidine Group	Nalbuphine Group	p-value
Sensory Block Onset (min)	8.1 ± 1.2	10.3 ± 1.4	< 0.001
Motor Block Onset (min)	15.2 ± 1.6	22.4 ± 1.8	< 0.001
Sensory Block Duration (min)	597.4 ± 56.8	378.3 ± 49.5	< 0.001
Motor Block Duration (min)	482.5 ± 44.2	289.6 ± 41.3	< 0.001
Time to First Rescue Analgesia (min)	650 ± 78	412 ± 49	< 0.001
No. of Rescue Analgesics in 12 hrs.	2.63 ± 0.83	3.28 ± 0.99	< 0.05

**Figure 2:**
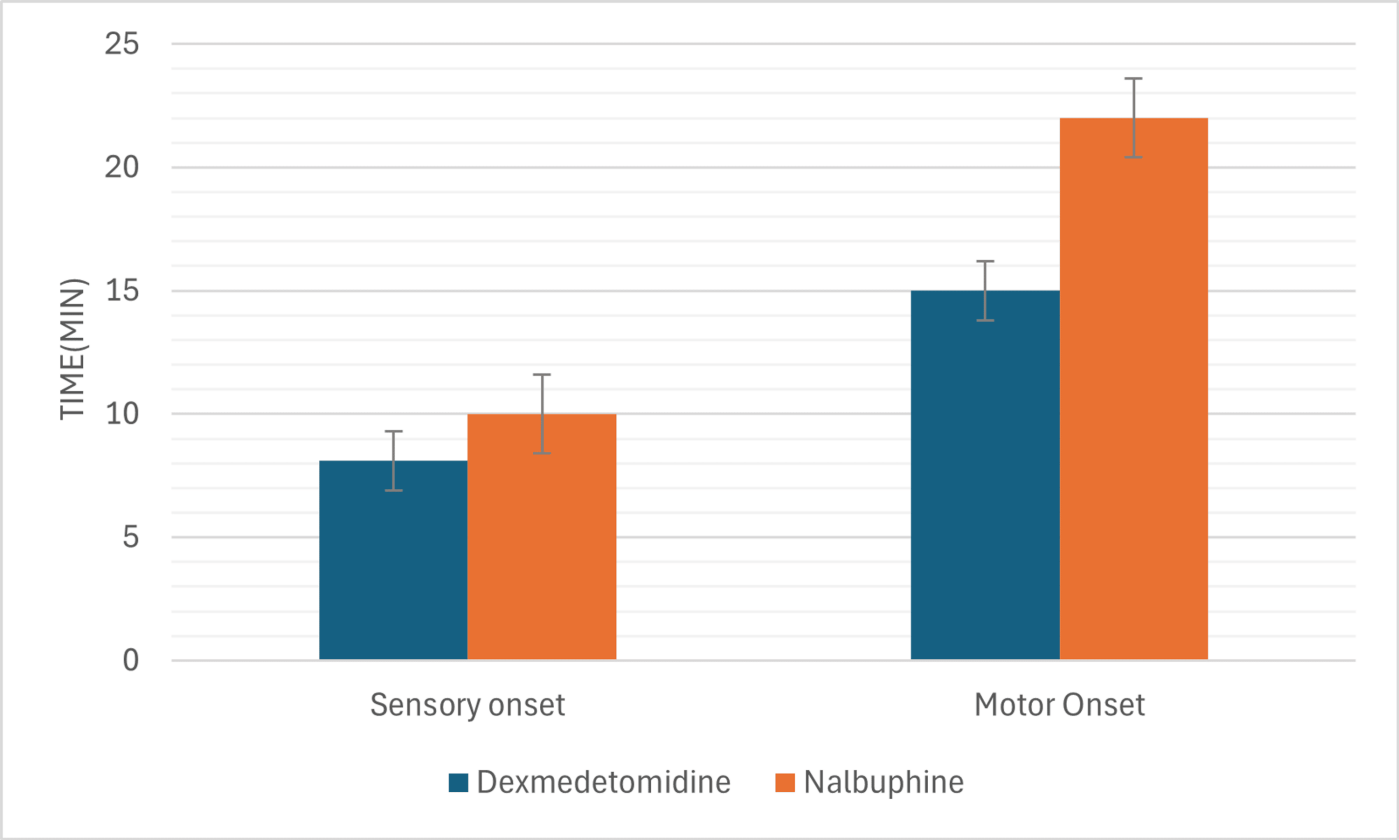
Onset of sensory and motor blocks in the two groups. Showing faster onset of both sensory and motor blocks in dexmedetomidine group

**Figure 3:**
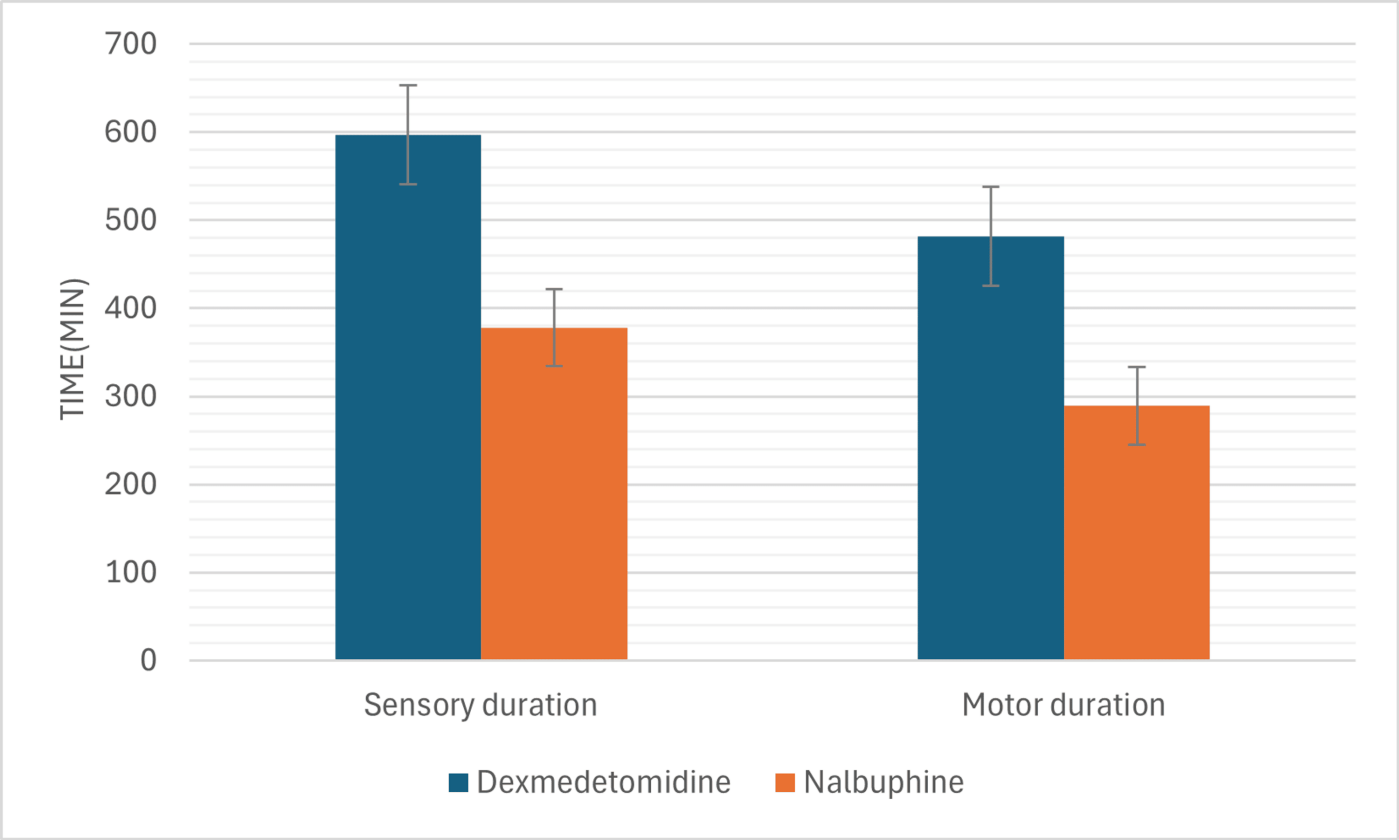
Duration of sensory and motor blocks in the two groups Showing significantly longer duration of sensory and motor blocks dexmedetomidine group.

The mean onset time for sensory block was 8.1 ± 1.2 minutes in Group D and 10.3 ± 1.4 minutes in Group N (p<0.05). For motor block, the mean onset was 15.2 ± 1.6 minutes in Group D and 22.4 ± 1.8 minutes in Group N (p<0.05) (Figure 2). The mean duration of sensory block was 597.4 ± 56.8 minutes in Group D compared to 378.3 ± 49.5 minutes in Group N (p<0.05). Similarly, the mean duration of motor block was 482.5 ± 44.2 minutes for Group D and 289.6 ± 41.3 minutes for Group N (p<0.05) (Figure 3).

**Figure 4:**
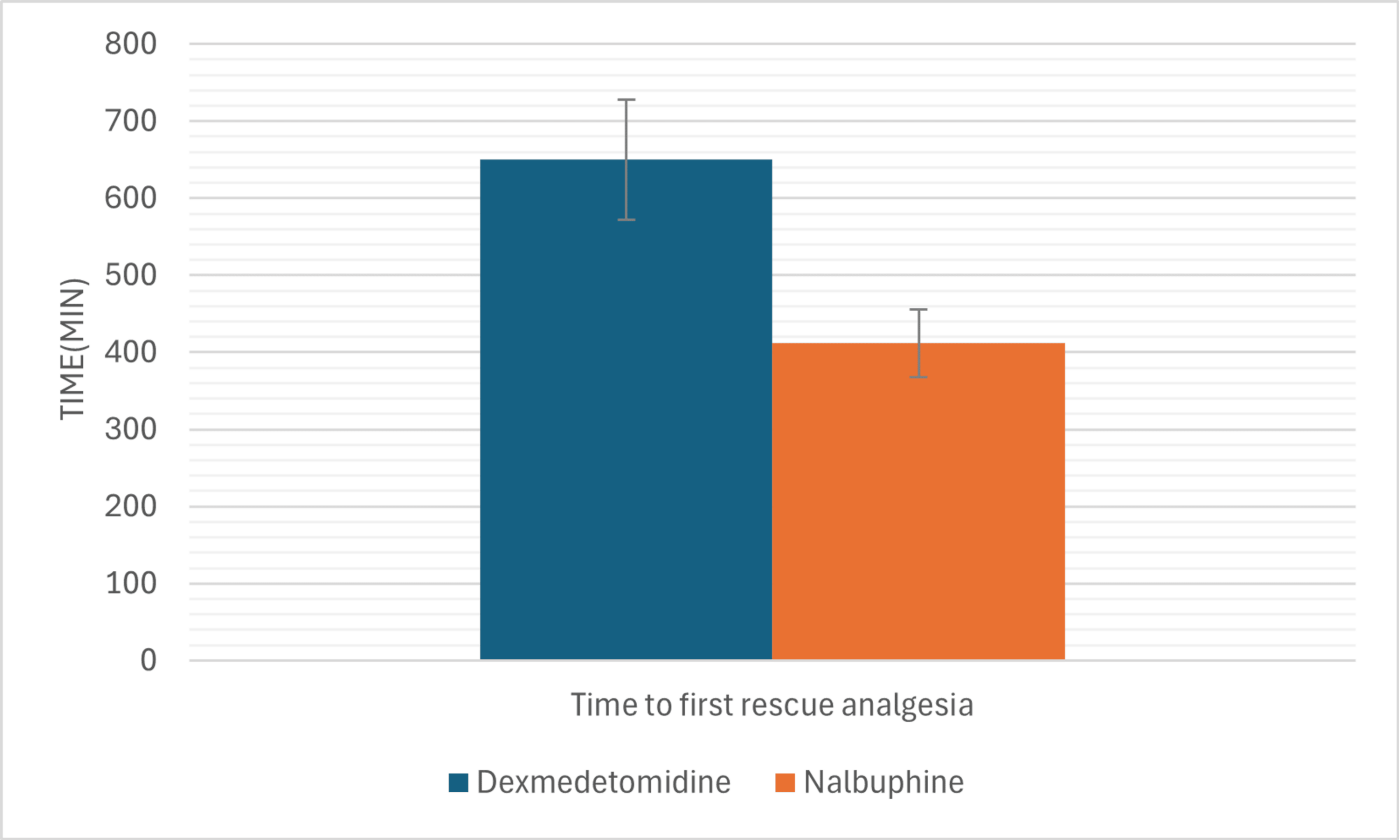
Time to first rescue and number of rescue analgesia in the two groups. Showing the dexmedetomidine group has a significantly longer duration of postoperative analgesia with fewer requirements of number of rescue analgesics.

The mean time for first rescue analgesia (Figure 4) was significantly longer in Group D (650 ± 78 minutes) compared to Group N (412 ± 49 minutes) (p<0.05). Furthermore, the mean number of rescue analgesia (injection tramadol 50 mg i.v.) required in the first 12 hours was significantly lower in Group D (2.63 ± 0.83) than in Group N (3.28 ± 0.99) (p<0.05). 

**Figure 5:**
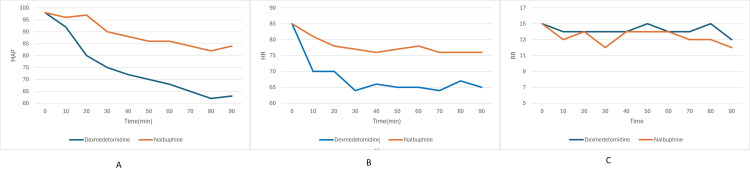
Hemodynamic variables in the two groups. Graph A depicts mean artery pressure (MAP), graph B depicts heart rate (HR), graph C depicts respiratory rate (RR). The dexmedetomidine group is showing significant hypotension and bradycardia while the respiratory rate in the two groups is comparable with no significant difference.

Mean arterial pressure showed a more pronounced drop in Group D compared to Group N over the first 90 minutes (Figure 5). The between-group differences were statistically significant at several time points (p<0.05). In heart rate a significant decline was observed in Group D starting from 10 minutes, becoming more pronounced from 30 minutes onwards (Figure 5). The difference was statistically significant from 10 minutes to 90 minutes (p<0.05). In contrast, the heart rate in Group N remained relatively stable throughout the observation period. Respiratory rate remained within normal limits for both groups throughout the observation period. However, a mild decline was noted in Group D, particularly beyond 30 minutes. The intergroup differences were not statistically significant at most time points (p > 0.05).

**Figure 6:**
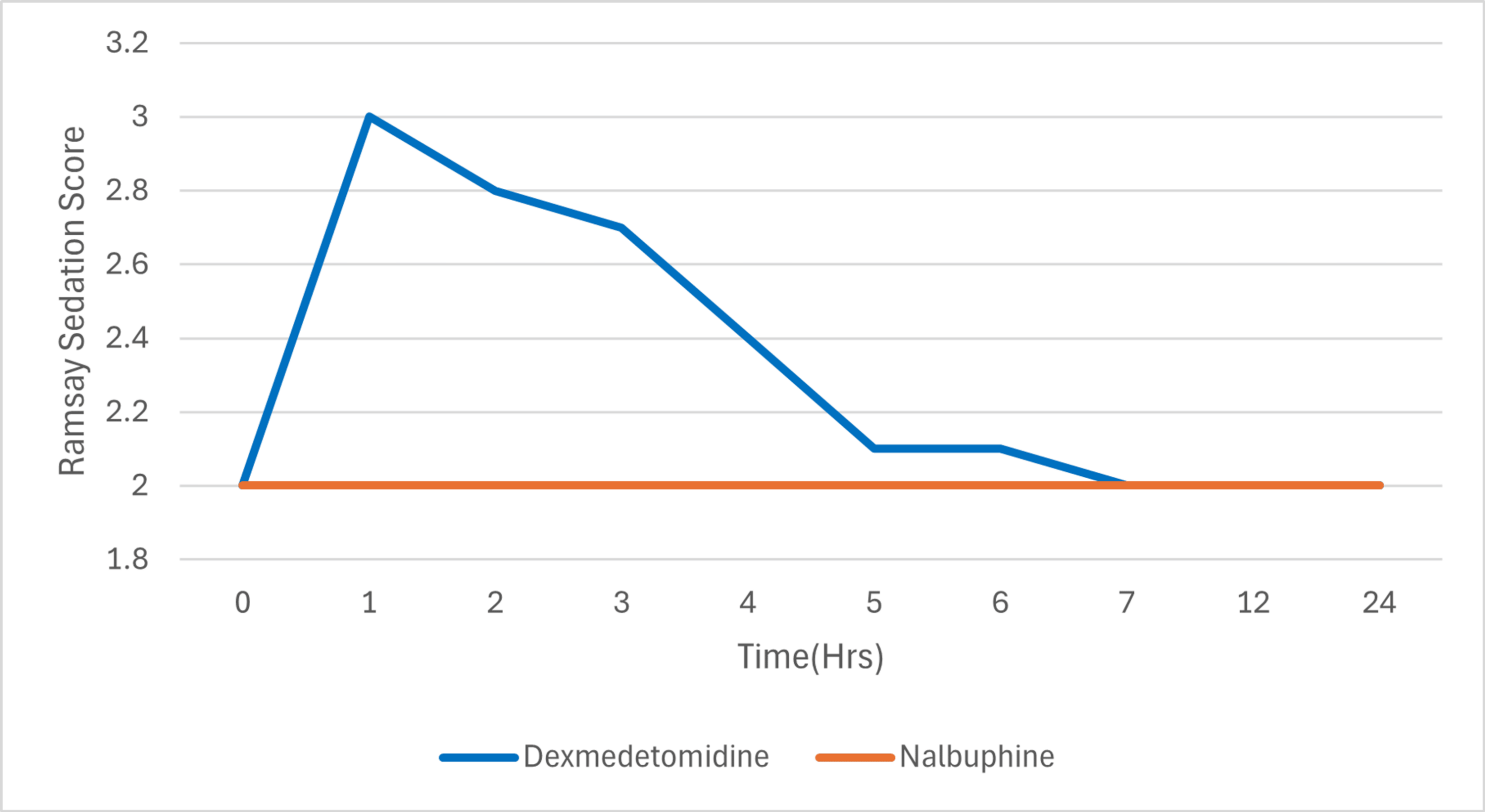
Ramsay sedation scores in the two groups. Showing significantly higher sedation scores in the dexmedetomidine group in the first four hours.

The Ramsay Sedation Scores (Figure 6) were consistently higher in Group D compared to Group N during the first four hours following block administration. The Ramsay Sedation Score peaked at 2.93 ± 0.66 at one hour in Group D, while it remained near baseline levels (1.93 ± 0.24) in Group N. Statistically significant differences (p < 0.05) were observed at one, two, three, and four hours. Beyond this period, the scores in both groups were comparable and not statistically significant (Figure 6).

**Figure 7:**
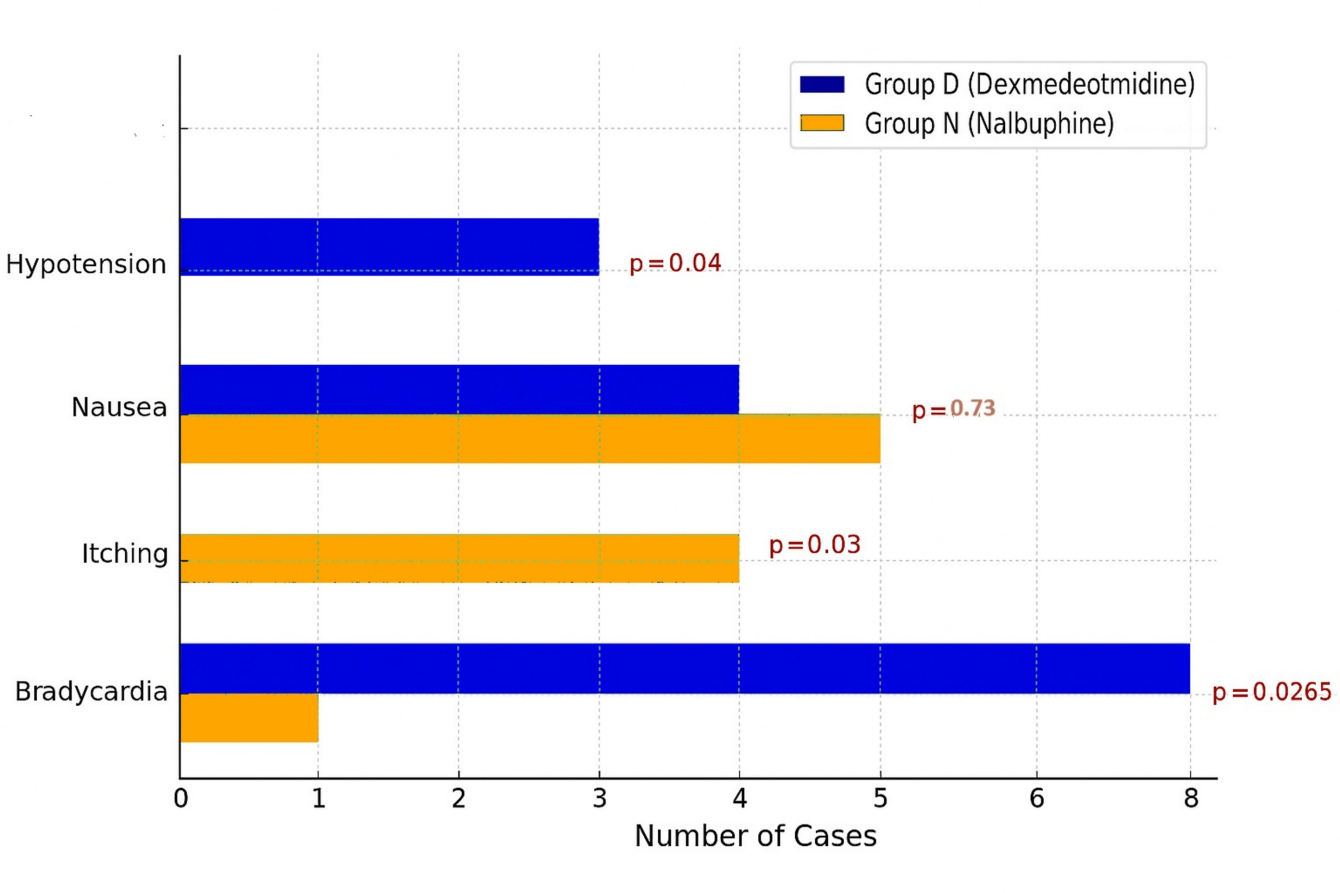
Complications in the two groups Showing the incidence of hypotension and bradycardia significantly more in dexmedetomidine group while the incidence of itching being significantly more in nalbuphine groups. Both groups have comparable incidence of nausea.

Considering complications, bradycardia (Figure 7) was observed in eight (25%) patients in Group D as compared to one (3%) patient in Group N (p<0.05). Episodes of bradycardia that were not accompanied by hypotension or any discomfort to the patient (n=6) were not treated with any medication. Only bradycardia episodes with hypotension (n=3), with two in Group D and one in Group N, were treated with injection mephentermine 6 mg i.v. bolus dose. Heart rate in all three cases responded to single dose of injection mephentermine within five minutes. Hypotension (Figure 7) was observed in three (10%) patients in Group D and none in Group N (p< 0.05). Hypotension was managed by giving injection mephentermine 6 mg i.v. bolus every five-minute interval if blood pressure was not responding. Blood pressure in all the cases responded within five to 10 minutes of injection mephentermine single (n=2) or maximum twice (n=1) i.v. bolus dose. Itching (Figure 7) was seen in four (15%) patients in Group N (15.6%) and none in Group D (p<0.05). The incidence of nausea (Figure 7) was comparable between the groups (18.8% vs. 12.5%; p = 0.73). No cases of pneumothorax or arterial puncture were observed in either group as the block was performed under ultrasonographic guidance.

## Discussion

This comparative analysis between dexmedetomidine and nalbuphine as adjuvants in brachial plexus blocks reveals distinct differences in block characteristics, analgesic requirements, sedation levels and hemodynamic effects.

The addition of dexmedetomidine to local anesthetics has been consistently shown to reduce the onset time for sensory and motor blocks while significantly prolonging their duration. In the current study, both sensory and motor block onset times were shorter in the dexmedetomidine group, with duration of blocks notably longer than those observed with nalbuphine. Similar findings have been reported by Gupta et al. [6], who demonstrated faster onset and prolonged duration of brachial plexus block with dexmedetomidine compared to opioids. In another study, Das et al. [7] found that dexmedetomidine enhanced the efficacy of ropivacaine in supraclavicular blocks, supporting its utility as an effective adjuvant.

The prolonged duration of analgesia observed in the dexmedetomidine group translated into a significantly delayed time to first rescue analgesia and a lower number of rescue analgesic requirements within 12 hours. This is consistent with studies such as those by Kathuria et al. [8], who reported superior postoperative analgesia with dexmedetomidine versus nalbuphine, and Bindra et al. [9], who observed prolonged analgesic effect and opioid-sparing benefits in the dexmedetomidine group.

Higher Ramsay Sedation Scores were consistently observed in the dexmedetomidine group during the early postoperative period, particularly in the first four hours. This sedative profile is advantageous for intraoperative anxiolysis but requires careful monitoring. Alansary et al. [10] emphasized dexmedetomidine's favorable sedative and analgesic synergy, although the risk of excessive sedation was noted. In contrast, nalbuphine, due to its kappa agonism and mu antagonism, provides analgesia with minimal sedation, as highlighted in comparative trials [11-13].

The present study revealed a higher incidence of bradycardia and hypotension in the dexmedetomidine group, aligning with its known alpha-2 agonist activity. Dexmedetomidine's ability to decrease sympathetic outflow leads to these cardiovascular side effects, as corroborated by various studies [14,15]. Meanwhile, nalbuphine demonstrated greater hemodynamic stability but with higher adverse effects of nausea, vomiting and pruritus, aligning with findings by previous studies [16,17], who advocated nalbuphine use in patients at risk of cardiovascular instability.

Limitations of this study include data from a single centre and exclusion of ASA 3 and 4 patients where dexmedetomidine may aggravate hypotension and bradycardia, so results can not be generalised to all patients. Multicentre trials with larger sample size, higher ASA class (3 or more) of the population and emergency cases are needed to validate these findings and improve generalisability.

## Conclusions

Both dexmedetomidine and nalbuphine are effective adjuvants for brachial plexus blocks. Dexmedetomidine provides faster onset, longer block duration, superior analgesia and higher sedation but at the cost of increased bradycardia and hypotension. Nalbuphine offers reasonable analgesia with stable hemodynamics and minimal sedation but with more nausea and pruritis. Clinicians should individualize adjuvant choice based on patient comorbidities - specifically cardiovascular ones - anesthetic goals and procedural context.
